# A prospective clinical trial of the second‐look procedure for transoral surgery in patients with T1 and T2 laryngeal, oropharyngeal, and hypopharyngeal cancer

**DOI:** 10.1002/cam4.2588

**Published:** 2019-10-08

**Authors:** Goshi Nishimura, Daisuke Sano, Yasuhiro Arai, Takashi Hatano, Hideaki Takahashi, Teruhiko Tanabe, Takashi Wada, Daiki Morishita, Nobuhiko Oridate

**Affiliations:** ^1^ Department of Otorhinolaryngology, Head and Neck Surgery School of Medicine Yokohama City University Yokohama Japan

**Keywords:** early T‐stage head and neck cancer, locoregional control, predictive factors, second‐look procedure, transoral surgery

## Abstract

**Background:**

Transoral surgery (TOS) has been widely applied for early T‐stage head and neck cancer (HNC). The resection is performed with a minimum safety margin for function preservation under a limited surgical field; therefore, it is difficult to have a strong conviction about the complete resection. This study aims to evaluate the completeness of the initial TOS procedure; possibility of primary control by TOS alone; and predictive factors in patients with early T‐stage laryngeal, oropharyngeal, and hypopharyngeal cancer.

**Methods:**

Patients were treated by TOS at the primary site with or without neck dissection. The patients were divided into two groups based on the pathological evaluation of their surgical specimens: the control (observation) group, in that the resection was considered complete and the intervention (second‐look procedure) group, in that incomplete tumor resection was suspected. The predictive factors for the possibility and/or limitations of complete resection by TOS were then analyzed.

**Results:**

The study enrolled 26 and 25 patients in the control and intervention group, respectively. The success rate for single resection was 66% and the predictive factor was tumor depth obtained by enhanced computed tomography (CT) examination (odds ratio, 7.870, *P* = .0243). The success rate for definitive therapy by TOS alone was 83% and the predictive factor was poor differentiation observed on pathological examination (odds ratio, 6.800, *P* = .0248).

**Conclusions:**

TOS has the potential for both definitive resection and function preservation with minimal invasiveness. Identification of the risk factors for TOS is advantageous for accurate treatment selection in patients with early T‐stage HNC.

## Graphical abstract

Transoral surgery has the potential for both definitive resection and function preservation with minimal invasiveness. Identification of the risk factors for transoral surgery, such as tumor depth obtained by enhanced CT examination and poor differentiation observed on pathological examination, is advantageous for accurate treatment selection in patients with early T‐stage head and neck cancer.

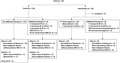

## 1 INTRODUCTION

The treatment of head and neck cancer (HNC) demands attention to both definitive curability and function preservation, for example, phonation and deglutition. Although most HNC patients present with advanced‐stage disease on their first visit, the number of early T‐stage patients has increased following the advent development of advanced diagnostic techniques such as positron emission tomography‐computed tomography (PET‐CT)^1^ and narrow‐band imaging (NBI) endoscopy,^2^ as well as because of an increased awareness of HNC among doctors in other fields.^3^ The development of surgical support instruments has promoted innovations in the field of minimally invasive surgery for the treatment of HNC, particularly with regard to definitive surgery in early T‐stage patients.^4^, ^5^, ^6^, ^7^, ^8^ Transoral surgery (TOS) is believed to derive equivalent outcomes as those of conventional (chemo‐)radiotherapy or open surgery; for example, total or partial laryngectomy, pharyngectomy.^4^, ^6^, ^9^, ^10^ However, one of the main concerns associated with TOS is regarding the achievement of a complete resection, given the limited surgical field and lack of maneuverability, as well as the minimum safety margin required for maximum function preservation.^11^ therefore, this study sought to identify the completeness or limitations of TOS through the application of the second‐look procedure in cases of suspected incomplete tumor resection.

## 2 MATERIALS AND METHODS

### 2.1 Study setting

A single institute (academic hospital), parallel two‐arm open‐label nonrandomized trial.

### 2.2 Endpoints

The primary endpoints were the evaluation of the completeness of the initial TOS resection (primary EP 1), and the possibility of primary control by TOS alone, allowing for repeat procedures as salvage surgeries in cases of suspected incomplete tumor resection in the initial TOS (primary EP 2). The secondary endpoints were overall survival (OS; the event is death; secondary EP 1), disease‐free survival (DFS; the events being the uncontrollability of existing cancer characterized by locoregional remnant, locoregional recurrence, and/or distant metastasis, appearance of new primary cancer, and death; secondary EP 2), and function‐preserving survival (FPS; the events being total laryngectomy and/or pharyngectomy as salvage surgical strategies and death; secondary EP 3).

### 2.3 Eligibility criteria

HNC is classified according to the 7th edition of the TNM classification.^12^ The present study enrolled patients with clinical T1 and T2 stage laryngeal, oropharyngeal, and hypopharyngeal carcinoma.

#### 2.3.1 Inclusion criteria

The eligibility criteria for enrollment of the patients in the trial included the following: pathologically proven carcinoma; primary tumor located in the larynx, oropharynx, or hypopharynx; cT1 and cT2 tumor on visual and endoscopic examinations and imaging, such as CT and/or magnetic resonance imaging (MRI); cN stage evaluated by ultrasonic echo (US echo) and/or PET‐CT; primary site assessed as resectable by TOS and regional lymph node by neck dissection on CT, MRI and/or US; no distant metastasis on PET‐CT (cM0); no prior treatment for any HNC; patients age above 20 years (legally adult in Japan); performance status (PS) of 0‐2 in compliance with Eastern Cooperative Oncology Group (ECOG) criteria; no contraindications for surgery under general anesthesia; and provision of written informed consent.

#### 2.3.2 Exclusion criteria

The exclusion criteria require the patients to be devoid of any of the following conditions: incurable synchronous malignancies, priority systemic diseases, and refusal to undergo the second‐look procedure. Patients who had previous malignancies were included in case these diseases were cured or well‐controlled (maintaining a complete response).

### 2.4 Enrollment and scheduled analysis

Patient enrollment started on 1 January 2014, and the scheduled duration of study was 2 years. The target sample size was 40‐60 patients, based on the average patient number (20‐30 patients per year) in our institution and the 2‐year entry period. We planned an interim analysis at the end of the entry period. If the number of patients enrolled reached the target number at the time of the interim analysis, enrollment would cease. If not, the enrollment would be extended until at least 40 patients are enrolled. The final analysis was to be performed 3 years after the last entry.

### 2.5 Treatment methods

#### 2.5.1 Surgery

Primary resection was performed using the TOS technique. The mucosal lesion was confirmed by NBI endoscopy and stained with Lugol's solution. The horizontal safety margin was set at a distance of 1‐3mm from the border of the lesion. The vertical resection was performed in the submucosal layer. After resection, rapid pathological examination of the margin was performed on the horizontal and vertical sections. In cases with positive margins in the rapid pathological examination, additional resection was performed until a negative margin was confirmed. The resected specimen was stretched on a cork board to clarify the directions and was subsequently fixed with formalin for permanent pathological diagnosis. The wound in oropharyngeal and hypopharyngeal cancer patients was covered with a polyglycolic acid sheet. Neck dissection was performed at the same time in node‐positive patients.

#### 2.5.2 Control (observation) arm

The patients in whom the resection was assessed as complete with negative margins by both rapid and permanent pathological examinations received no additional therapy for local control.

#### 2.5.3 Intervention (second‐look procedure) arm

Contrary to our expectations, the patients in whom complete resection could not be not confirmed by permanent pathological examination, that is, cases with the presence of positive or close margins, were included in the intervention arm. Two to three months after the first TOS operation, the second‐look procedure (re‐TOS) was performed in these patients. The period between the first and second TOS were defined as the wound repair time. The precise observation in second‐look TOS and close follow‐up was applied in these patients during this period. During the second‐look procedure, precise observation by high‐vision endoscope, pathological examination from the resected primary site, and additional resection of tumor remnants were performed. In cases where the additional resection could not be completed by TOS, open surgery and/or (chemo‐)radiotherapy were administered as alternative definitive therapy.

#### 2.5.4 Follow‐up

All patients were followed‐up for at least 5 years after patient accrual was completed. Visual and endoscopic observations of the primary site were performed every month for the first and second years, and every 2‐3 months from the third to fifth years. Enhanced cervical CT and/or US for the primary site and regional lymph nodes was performed every 3‐6 months for the first and second years and every 6‐12 months from the third to fifth years. Finally, PET‐CT was performed every year for the first and second years and enhanced whole‐body CT was performed every year from the third to fifth years for the evaluation of distant metastasis.

### 2.6 Statistical analysis

Fisher's exact test and a Cox proportional hazards model were used for univariate and multivariate comparisons, respectively. The outcome variables were the completeness of the first TOS resection (primary EP 1), the possibility of local control by TOS (primary EP 2), and the survival endpoints (secondary EP 1‐3). The predictive variables were clinical characteristics (age, sex, primary site, TN stage, tumor shape, enhanced CT observation) and pathological characteristics (histological type, differentiation, margin study, lymphatic invasion, vascular invasion, nerve invasion). The Kaplan‐Meier method was used for evaluating the survival endpoints. The survival rates were statistically analyzed using Wilcoxon tests.

## 3 RESULTS

### 3.1 Trial status

The UMIN Clinical Trials Registry (UMIN000012485) was completed on 14 December 2013. Patient enrollment started on 1 January 2014 and enrollment closed on 31 March 2016, with 51 patients. The observation period ended on 31 March 2018.

### 3.2 Patient characteristics and examination results

**Figure 1:**
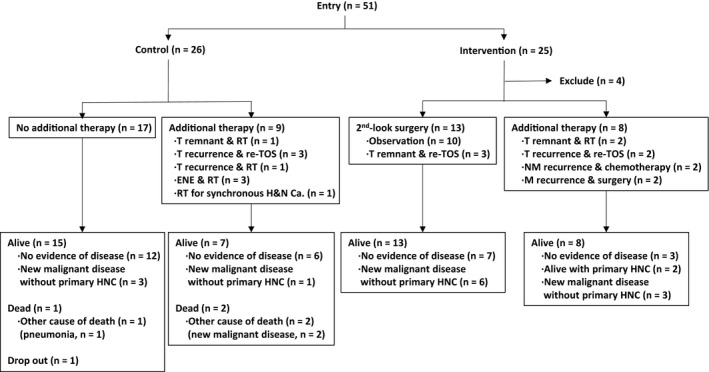
Diagram of patient therapeutic courses. Fifty‐one patients were enrolled, with 26 and 25 patients in the control and intervention groups, respectively. Four patients were excluded from the intervention group; therefore, a total of 21 patients were analyzed for intervention. Abbreviation: ENE, extranodal extension; HNC, head and neck cancer; M, distant metastasis; N, regional lymph node; RT, radiotherapy; T, primary tumor site; TOS, transoral surgery

The patient clinical characteristics are summarized in Table 1. The control and intervention groups included 26 and 25 patients, respectively. There were no significant differences between these groups by Fisher's exact tests. Four patients were excluded from the intervention group after entry for the following reasons; two patients refused to undergo second‐look procedures, one patient had a poor general status for operation, and one patient dropped out. The patients' therapeutic courses are shown diagrammatically in Figure 1. Within the TOS, we applied transoral videolaryngoscopic surgery (TOVS)^6^, ^8^, ^10^ for 34 patients (14 of laryngeal cancer, 13 of oropharyngeal cancer, and 7 of hypopharyngeal cancer), and endoscopic laryngopharyngeal surgery (ELPS)^5^, ^7^ for 13 patients (all hypopharyngeal cancer). The follow‐up periods ranged from 4‐63 months (mean, 42 months) in the control group and 36‐61 months (mean, 48 months) in the intervention group. The success rates of completeness of the initial TOS resection (primary EP 1) and primary control by TOS alone (primary EP 2) were 66% (in 31 of 47 patients) and 83% (in 39 of 47 patients), respectively.

**Table 1:** Enrolled patients' clinical characteristics

	Control (n = 26)	Intervention (n = 25)
Age (mean)	48‐85 (67)	55‐87 (71)
Sex		
Male	24	22
Female	2	3
Primary		
Larynx	9	5
Oropharynx	10	5
Hypopharynx	7	15
T		
T1	15	9
T2	11	16
N		
N0	21	24
N1	1	0
N2	4	1

Note

Twenty six patients were divided into the control (observation) group and 25 patients were divided into the intervention (second‐look procedure) group. There was no significant difference about the patient backgrounds between these two groups by Fisher's exact test.

The examination results of the analyzed patients are summarized in Table 2. Enhanced CT was performed in 37 patients and the cut‐off values were defined by the receiver operating characteristic (ROC) curve between the clinical results (primary EP 1; completeness of resection in the initial TOS, primary EP 2; primary control by TOS alone, secondary EP 1; OS, secondary EP 2; DFS, secondary EP 3; and FPS) and tumor size (length and depth). The areas under the ROC curves (AUCs) and 95% confidence intervals (CIs) were as follows; 0.659 and 0.477‐0.841 for length and 0.701 and 0.522‐0.881 for depth in primary EP 1; 0.659 and 0.477‐0.841 for length and 0.701 and 0.522‐0.881 for depth in primary EP 2; 0.758 and 0.496‐1.000 for length and 0.803 and 0.621‐0.985 for depth in secondary EP 1; 0.580 and 0.392‐0.769 for length and 0.619 and 0.437‐0.800 for depth in secondary EP 2; and 0.758 and 0.496‐1.000 for length and 0.803 and 0.621‐0.985 for depth in secondary EP 3.

**Table 2:** The examination results of analyzed patients

	Control (n = 26)	Intervention (n = 21)
Clinical feature		
Age (mean	48‐85 (67)	55‐84 (71)
Sex		
Male	24	18
Female	2	3
Primary		
Larynx	9	5
Oropharynx	10	3
Hypopharynx	7	13
T		
T1	15	8
T2	11	13
N		
N0	21	21
N1	1	0
N2	4	0
Tumor shape		
Flat or ulcer	10	13
Balky	16	8
Imaging examination		
Enhanced CT examination	(n = 21)	(n = 16)
Primary EP 1		
Length ≥7 mm	11	8
Depth ≥7 mm	6	2
Primary EP 2		
Length ≥7 mm	11	8
Depth ≥7 mm	6	2
Secondary EP 1		
Length ≥8 mm	10	8
Depth ≥3 mm	10	5
Secondary EP 2		
Length ≥2 mm	13	9
Depth ≥1 mm	13	9
Secondary EP 3		
Length ≥8 mm	10	8
Depth ≥3 mm	10	5
Submucosal invasion		
Positive	10	6
Negative	11	10
Pathological examination		
Pathology		
Squamous cell carcinoma	25	21
Spindle cell carcinoma	1	0
Differentiation		
Carcinoma in situ	5	4
Poorly	5	4
Moderately	7	8
Well	7	4
Unclassified	2	1
Lymphatic invasion		
Positive	2	1
Negative	24	20
Vascular invasion		
Positive	5	0
Negative	21	21
Nerve invasion		
Positive	0	0
Negative	26	21
Surgical margin		
Positive or suspicious	6	11
Negative	20	10

Note

The predictive factors were divided into three main groups, that is, clinical characteristics, enhanced CT features, and pathological evaluations. The cut‐off values of tumor length and depth were calculated by ROC curves in each endpoints.

### 3.3 Statistical analyses

#### 3.3.1 Univariate and multivariate analysis

The results of the univariate analysis are summarized in Table 3. In primary EP1, the significant predictive factors (*P* < .05) were N stage of N0 or N2 and depth in CT. In primary EP 2, these factors were N stage of N0 or N2, length or depth in CT, and poor differentiation on pathological evaluation. In secondary EP 1, these factors were depth in CT and vascular invasion in pathological evaluation. In secondary EP 2, there were no significant differences in predictive factors. In secondary EP 3, the significant predictive factors were depth in CT and vascular invasion in pathological evaluation.

**Table 3:** The results of univariate analysis in each endpoints by Fisher's exact test

Predictive factors	Primary EP 1			Primary EP 2			Secondary EP 1			Secondary EP 2			Secondary EP 3		
	Success (n)	failure (n)	*P* value	Success (n)	failure (n)	*P* value	Success (n)	failure (n)	*P* value	Success (n)	failure (n)	*P* value	Success (n)	failure (n)	*P* value
Age (median; 68 y)															
<68	13	8	.758	15	6	.115	18	3	.082	10	11	1	18	3	.082
≥68	18	8		24	2		26	0		13	13		26	0	
Sex															
Male	28	14	1	35	7	1	39	3	1	21	21	1	39	3	1
Female	3	2		4	1		5	0		2	3		5	0	
Primary															
Larynx	10	4	.742	13	1	.405	14	0	.544	9	5	.212	14	0	.544
Oropharynx	8	5	.739	11	2	1	11	2	.181	5	8	.517	11	2	.181
Hypopharynx	13	7	1	15	5	.258	19	1	1	9	11	.77	19	1	1
TN classification															
T1	16	7	.76	21	2	.245	22	1	1	12	11	.773	22	1	1
T2	15	9	.76	18	6	.245	22	2	1	11	13	.773	22	2	1
N0	31	11	.00285	39	3	.0000365	40	2	.292	21	21	1	40	2	.292
N1	0	1	.34	0	1	.17	1	0	1	1	0	.489	1	0	1
N2	0	4	.0102	0	4	.000392	3	1	.239	1	3	.609	3	1	.239
Tumor shape															
Flat or ulcer	17	7	.547	19	4	1	22	1	1	10	13	.564	22	1	1
Balky	14	9		20	4		22	2		13	11		22	2	
Length in CT															
<cut‐off value	14	4	.0911	18	0	.00309	18	1	.595	10	5	.184	16	2	.604
≥cut‐off value	9	10		11	8		16	2		9	13		18	1	
Depth in CT															
<cut‐off value	21	8	.0345	26	3	.0056	22	0	.0586	10	5	.184	12	3	.0586
≥cut‐off value	2	6		3	5		12	3		9	13		22	0	
Submucosal invasion in CT															
Positive	9	7	.733	10	6	.0554	14	2	.568	7	9	.515	14	2	.568
Negative	14	7		19	2		20	1		12	9		20	1	
Pathology															
Squamous cell carcinoma	31	15	1	39	7	1	43	3	1	22	24	1	43	3	1
Spindle cell carcinoma	0	1		0	1		1	0		1	0		1	0	
Differentiation															
Carcinoma in situ	6	3	1	9	0	.323	9	0	1	4	5	1	9	0	1
Poorly	5	4	.466	5	4	.0331	7	2	.0895	3	6	.461	7	2	.0895
Moderately	10	5	1	14	1	.406	15	0	.541	9	6	.359	15	0	.541
Well	8	3	.725	9	2	1	10	1	.56	5	6	1	10	1	.56
Unclassified	2	1	1	2	1	.436	3	0	1	2	1	.609	3	0	1
Lynphatic invasion															
Positive	2	1	1	2	1	.436	2	1	.183	2	1	.609	2	1	.183
Negative	29	15		37	7		42	2		21	23		42	2	
Vascular invasion															
Positive	3	2	1	3	2	.196	3	2	.0265	2	3	1	3	2	.0265
Negative	28	14		26	3		41	1		21	21		41	1	
Nerve invasion															
Positive	0	0	1	0	0	1	0	0	1	0	0	1	0	0	1
Negative	31	16		39	8		44	3		23	24		44	3	
Surgical margin															
Positive or suspicious	10	7	.528	12	5	.118	15	2	.544	6	11	.227	15	2	.544
Negative	21	9		27	3		29	1		17	13		29	1	

Note

Statistical significant *P* value was defined less than .05.

Multivariate logistic regression analysis was performed to examine the relationships between clinical response and the results of univariate analysis (Table 4). The results revealed that the depth in CT (odds ratio: 7.87, 95% CI: 1.31‐47.4, *P* = .0243) was an independent significant predictive factor in primary EP1, while poor differentiation by pathological examination (odds ratio: 6.38, 95% CI: 1.28‐d36.3, *P* = .0248) was an independent significant predictive factor in primary EP 2. There was no significant predictive factor of the secondary endpoints.

**Table 4:** The results of multivariate analysis by Cox proportional hazards model

	Odds ratio	95% CI	*P* value
Primary EP 1			
Depth in CT	7.87	1.31‐47.4	.0243
Primary EP 2			
Poorly differentiation	6.8	1.28‐36.3	.0248

Note

Independent significant predictive factors were tumor depth by enhanced CT in primary endpoint 1 and poorly differentiation by pathological examination in primary endpoint 2. There was no independent significant predictive factor in secondary endpoints.

#### 3.3.2 Survival

**Figure 2:**
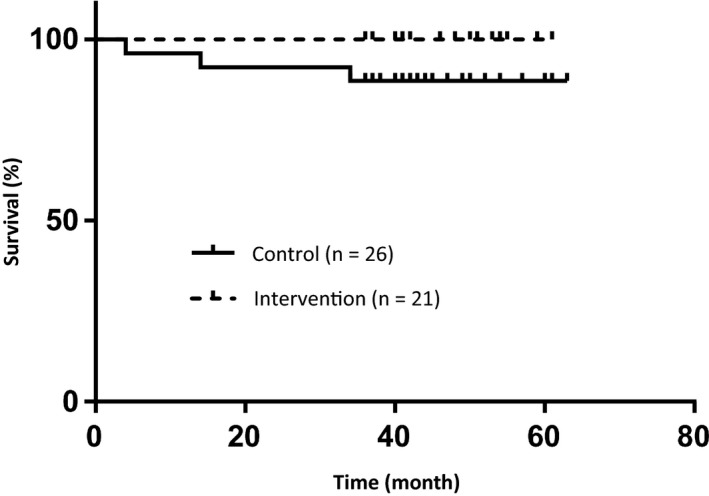
Overall survival (OS) was shown by the Kaplan‐Meier method. The 5‐year OS in the control and intervention groups were 88% and 100%, respectively, and 94% in all patients. There was no significant difference (*P* = .11) between the control and intervention groups

**Figure 3:**
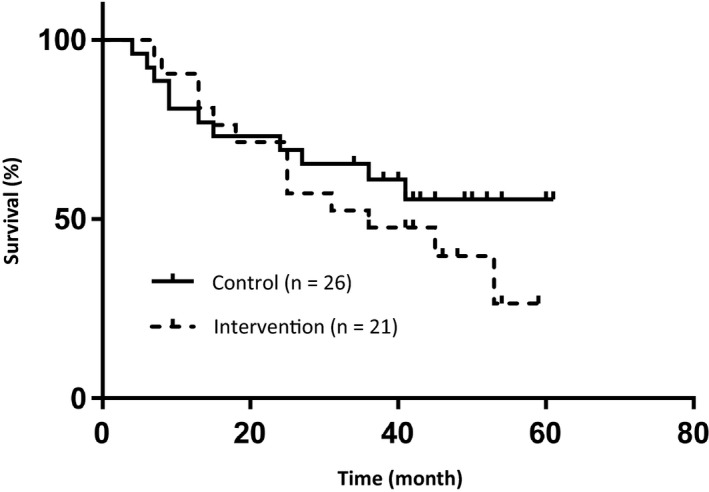
Disease‐free survival (DFS). The 5‐year DFS in the control group and intervention groups were 55% and 26%, respectively, and 40% in all patients. There was no significant difference (*P* = .63) between the control and intervention groups

**Figure 4:**
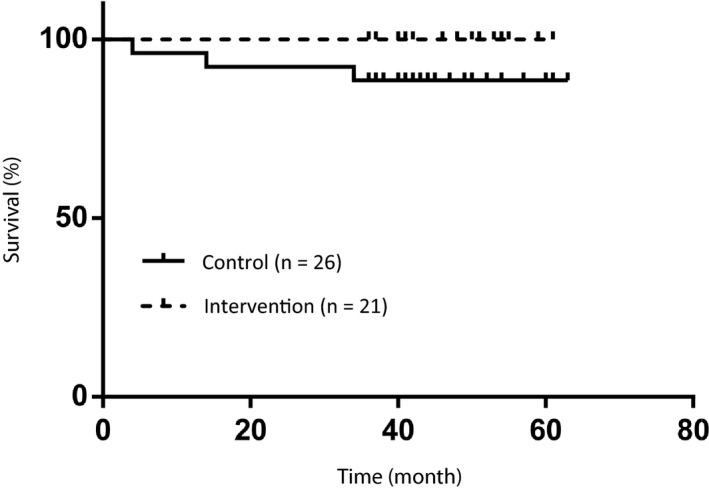
Function‐preserving survival (FPS). The 5‐year FPS in the control group and intervention groups were 88% and 100%, respectively, and 94% in all patients. There was no significant difference (*P* = .11) between the control and intervention groups

The 5‐year OS in the control and intervention groups were 88% and 100%, respectively (Figure 2), and 94% in all patients. There was no significant difference (*P* = .11) between the control and intervention groups. No patient died of a primary HNC. The 5‐year DFS in the control and intervention groups were 55% and 26%, respectively (Figure 3), and 40% in all patients. There was no significant difference (*P* = .63) between the control and intervention groups. The events were newly diagnosed malignant disease (n = 13), recurrence of the primary HNC (n = 10), and other causes of death (n = 1). Including overlapping events, 15 patients (32%, 15 of 47 patients) had newly diagnosed malignant disease during the follow‐up period, and that made the DFS to be worse comparing to the OS. There were no significant predictive factors in the univariate and multivariate analyses (cut‐off values were 5 mm in length and 1 mm in depth by CT evaluation, calculated from ROC between the newly diagnosed malignant disease and tumor size). The 5‐year FPS in the control group and intervention groups were 88% and 100%, respectively (Figure 4), and 94% in all patients. There was no significant difference (*P* = .11) between the control and intervention groups. No patient received radical surgery as salvage treatment.

## 4 DISCUSSION

TOS includes TOVS,^6^, ^8^, ^10^ transoral LASER microsurgery (TLM),^13^ transoral robotic surgery (TORS),^4^ and ELPS.^5^, ^7^ TOS is considered a definitive therapy, with comparable outcomes as radiotherapy or open surgery, for early T‐stage laryngeal, oropharyngeal, and hypopharyngeal cancer, consistent with organ and function preservation. TOS is performed with neck dissection in node‐positive patients with early T‐stage disease. Previous reports indicated a 5‐year recurrence‐free survival (RFS) of 85.3% and 10‐year OS of 74.7% by TLM^14^; 5‐year cause‐specific survivals (CSS) of 95.3% for stage I and 96.0% for stage II by chemoradiotherapy^15^ in laryngeal cancer. The 3‐year RFS was 82% by TOS,^16^ and the 5‐year disease‐free survival was 77% by radiotherapy^17^ in oropharyngeal cancer. The 5‐year OS was 77.6% in pT and 52.8% in pT2, the 5‐year RFSs was 77.9% in pT1 and 74.6% in pT2, the 5‐year CSS was 96.3% in pT1 and 96.7% in pT2 by TLM^18^ and the 5‐year OS and CSS were 58% and 75% by radiotherapy,^19^ respectively, in hypopharyngeal cancer. Our findings indicating 94% 5‐year OS and FPS are similar to those of previous reports on early stage HNC.

There are merits and demerits associated with these transoral surgical procedures. Endoscope‐assisted surgeries, such as TOVS, TORS, and ELPS, afford precise views that allow resection to be completed with minimal invasiveness.^4^, ^5^, ^6^, ^7^, ^8^, ^10^ Straight operation field surgeries such as TOVS and TLM provide easy maneuverability in a similar manner to that of the laryngo‐microsurgery technique,^6^, ^8^, ^9^, ^10^ while TORS allows a better view and increased precision, especially when working in less‐accessible areas.^4^ The common demerits of these techniques include the limited view and working field.^4^, ^5^, ^6^, ^7^, ^8^, ^9^, ^10^ TOS aims for definitive resection with minimum safety margins that may contribute to organ and function preservation. However, this approach increases the risk of remnant tumor. Although complete resection may be confirmed on pathological examination, the decision‐making is difficult because the surgical margin is kept to a minimum and the specimen tends to shrink during electrical coagulation and formalin fixation. The difficulty is compounded by the requirement for rapid pathological diagnosis during the operation, which may lead to challenges in identifying positive or close margins. These factors affected the primary site distribution in this study as well; the number of oropharyngeal cancer patients tended to increase in the control group and the number of hypopharyngeal cancer patients tended to increase in the intervention group, although the difference was not statistically significant. In TOS, the oropharynx may offer a better surgical view and field that may lead to complete resection of the tumors; however, the hypopharynx has a limitation in providing sufficient room for surgical maneuvering.

Salvage treatment for remnant and recurrent tumors at the primary site after first TOS differs by institution. The salvage treatment is selected from among a number of options including re‐TOS, radical dissection, and chemoradiotherapy depending on the recurrence status.^7^, ^9^, ^13^, ^20^ Radical dissection is considered to be the most reliable and effective treatment method despite the loss of function, such as aphonia, dysarthria, and dysphagia, in some cases. Although chemoradiotherapy can preserve organs, sensory torpor and/or radiation scars cause functional disorders; for example, hoarseness and dysphagia.^21^, ^22^ Moreover, radical dissection after chemoradiotherapy failure indicates a high incidence of postoperative complications.^23^ Taken together, the demerits associated with radical dissection and/or chemoradiotherapy and the concept of function preservation that underpins TOS compel us to select re‐TOS as our first‐choice salvage treatment. Early detection of remnant and recurrent tumors is necessary when considering re‐TOS. Recurrence after complete resection is stochastic and it is impossible to absolutely prevent such recurrences. However, second‐look TOS can allow disease control by use of the TOS technique with function preservation. We hypothesize that one of the causes of a remnant tumor is the limited scope of observation and resection that results from the restricted surgical field view and maneuverability. Second‐look TOS is adopted for high‐risk patients with remnant tumor, and precise observation and/or biopsy is performed for the confirmation of a pathological negative status in cases without remnant tumor, while additional resection is performed for truly remnant cases. The success rate of completeness of resection by initial TOS (primary EP 1) was 66% (31/47), and primary control by TOS alone (primary EP 2) was 83% (39/47). Salvage re‐TOS was performed in eight patients, three of whom were salvaged by the second‐look procedure and found to be benefitted (23%; 3 of the 13 second‐look operation patients). The adequacy about the probability needs to be discussed to continue this second‐look procedure in the future. The risk factors identified in this study, that is, tumor depth by enhanced CT and poor differentiation in pathological examination, help our discussion, in that, they may be elementary for the prediction of tumor remnants and/or recurrence by initial TOS, and attention must be paid to such patients preserving with these risk factors.

Although our treatment protocol needs burden on patients who are divided in second‐look TOS group, we consider this strategy has many advantages; we can have strong conviction to the complete resection by the initial TOS for no remnant tumor patients; we can perform re‐TOS at the second‐look TOS for local remnant tumor patients; we can proceed other treatment strategy, such as radiotherapy and open surgery, for the patients who cannot resect the remnant tumor by TOS technique immediately after the second‐look TOS.

Moreover, previous report^24^ indicated that the percentage of second primary malignant tumors exceeded that of the primary HNC recurrences, 4 years after the diagnosis of the primary tumor in advanced‐stage cases. Similar attention is required during the follow‐up period in early stage HNC patients.

## 5 CONCLUSION

Our findings suggest that TOS may be considered as a minimally invasive treatment and allows function preservation in patients with early T‐stage laryngeal, oropharyngeal, and hypopharyngeal cancer. The risk factors for local remnant and recurrence by TOS include tumor depth by enhanced CT and poor differentiation by pathological examination.

## CONFLICT OF INTEREST

We have no conflict of interest.

## AUTHOR CONTRIBUTIONS

Goshi Nishimura involved in conceptualization, data curation, formal analysis, methodology, investigation, project administration, validation, writing–original draft, and writing–review and editing. Daisuke Sano, Yasuhiro Arai, Takashi Hatano, Hideaki Takahashi, Teruhiko Tanabe, Takashi Wada, and Daiki Morishita involved in methodology, supervision, and writing–review and editing. Nobuhiko Oridate involved in conceptualization, methodology, supervision, and writing–review and editing.

## LIMITATIONS

This study was conducted at a single institution with a limited number of patients.

## ETHICS APPROVAL AND CONSENT TO PARTICIPATE

Ethical approval for the study was obtained from the Yokohama City University Institutional Review Board (#B131107003). Written informed consent was obtained from the study participants for the publication of their data.

## Data Availability

The data that support the findings of this study are available on request from the corresponding author. The data are not publicly available due to privacy or ethical restrictions.
